# Patch-based convolutional neural networks for automatic landmark detection of 3D facial images in clinical settings

**DOI:** 10.1093/ejo/cjae056

**Published:** 2024-11-28

**Authors:** Bodore Al-baker, Ashraf Ayoub, Xiangyang Ju, Peter Mossey

**Affiliations:** Orthodontic Department, Hamad Dental Center, Hamad Medical Corporation, Doha, Qatar; Scottish Craniofacial Research Group, Glasgow University Dental Hospital & School, School of Medicine, College of Medical, Veterinary and Life Sciences, University of Glasgow, Glasgow, United Kingdom; Medical Devices Unit, Department of Clinical Physics and Bioengineering, National Health Service of Greater Glasgow and Clyde, Glasgow, United Kingdom; Dental Hospital and School, University of Dundee, Dundee, United Kingdom

**Keywords:** 3D facial images, landmark annotation, convolutional neural networks, orthodontics, orthognathic surgery, mean localization error

## Abstract

**Background:**

The facial landmark annotation of 3D facial images is crucial in clinical orthodontics and orthognathic surgeries for accurate diagnosis and treatment planning. While manual landmarking has traditionally been the gold standard, it is labour-intensive and prone to variability.

**Objective:**

This study presents a framework for automated landmark detection in 3D facial images within a clinical context, using convolutional neural networks (CNNs), and it assesses its accuracy in comparison to that of ground-truth data.

**Material and methods:**

Initially, an in-house dataset of 408 3D facial images, each annotated with 37 landmarks by an expert, was constructed. Subsequently, a 2.5D patch-based CNN architecture was trained using this dataset to detect the same set of landmarks automatically.

**Results:**

The developed CNN model demonstrated high accuracy, with an overall mean localization error of 0.83 ± 0.49 mm. The majority of the landmarks had low localization errors, with 95% exhibiting a mean error of less than 1 mm across all axes. Moreover, the method achieved a high success detection rate, with 88% of detections having an error below 1.5 mm and 94% below 2 mm.

**Conclusion:**

The automated method used in this study demonstrated accuracy comparable to that achieved with manual annotations within clinical settings. In addition, the proposed framework for automatic landmark localization exhibited improved accuracy over existing models in the literature. Despite these advancements, it is important to acknowledge the limitations of this research, such as that it was based on a single-centre study and a single annotator. Future work should address computational time challenges to achieve further enhancements. This approach has significant potential to improve the efficiency and accuracy of orthodontic and orthognathic procedures.

## Introduction

The landmark annotation of 3D facial images in clinical settings is of particular importance in orthodontics and orthognathic surgeries for the accurate analysis of facial morphology, including identifying linear and angular facial measurements. Furthermore, the utilization of dense correspondence analyses—a surface-based method—facilitates comparisons between a patient’s facial surface and that of unaffected individuals, enabling the monitoring of facial changes before and after treatment [1–3]. This is crucial for diagnosing and evaluating the outcomes of surgical correction for patients with facial deformities, as well as for planning and assessing treatments [2, 4–6].

Landmark identification is mostly carried out manually, which is time-consuming and labour-intensive. It also requires a high level of expertise and training to minimize the potential landmarking errors and inconsistencies [7]. Furthermore, manual landmarking is susceptible to personal biases and is dependent on the clinician’s level of experience. This could lead to inter-observer variability and potential diagnostic inaccuracies [8].

Automatic landmarking tools are valuable resources. However, their reliability relies on the accuracy of the algorithm employed to detect facial landmarks within the captured digital images. In addition, these tools can also increase the efficiency of the diagnostic process, allowing clinicians to analyse big data, which would ultimately lead to improved patient care.

In recent years, convolutional neural networks (CNNs) have emerged as a promising tool for facial landmark detection in computer vision [9]. CNNs use a powerful mathematical approach for deep learning that allows the analysis of complex patterns within 3D facial images. A CNN’s mechanism is based on convolving local receptive fields over the image and performing element-wise multiplication with learnable filters or kernels that allow the CNN to extract valuable features [10]. The interconnected layers of the CNN can then recognize patterns across different image regions. Therefore, a CNN is a suitable choice for facial landmark detection when a high level of accuracy is required.

Despite CNNs being extensively applied in computer vision, their application in clinical settings, particularly in orthodontics and orthognathic surgeries, remains limited [11]. This may be due to the challenges associated with collecting and annotating large amounts of high-quality clinical datasets for training and evaluating reliable deep-learning models [12, 13]. Given the scarcity of studies on the automated identification of soft-tissue landmarks in clinical 3D facial images, this study aims to develop and assess the accuracy of an automated method using the CNN approach for the automatic detection of landmarks in 3D facial images within clinical settings.

## Materials and methods

### Ethics statement

Ethical approval was obtained for this study (REC reference: 21/ES/0042). All procedures, including the filing and storage of data, adhered to the guidelines and policies set forth by health authorities.

### Method overview

Deep learning (DL) networks were employed for the task of facial landmark detection in 3D facial images. Each DL detection network was trained using databases containing manually annotated facial images. After training, these DL networks were utilized to automatically identify facial landmarks in a new set of previously unseen 3D facial images. CNNs were chosen specifically for this purpose.

**Figure 1.**
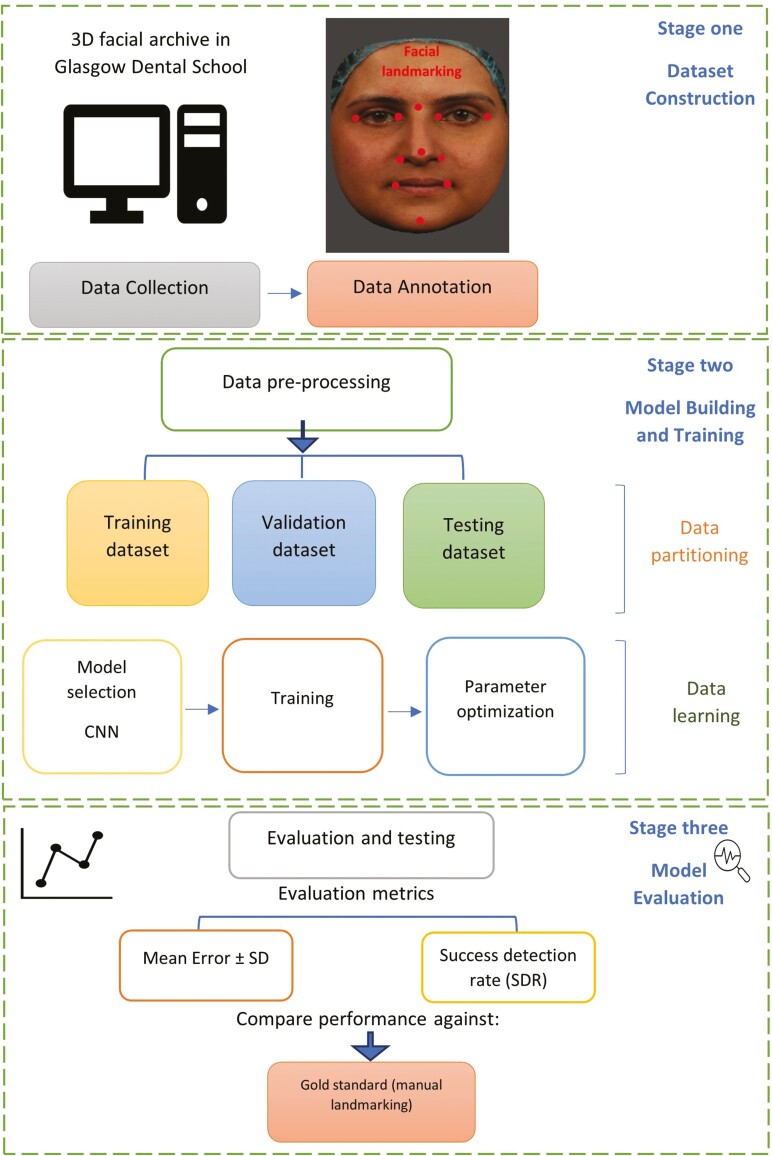
Workflow of dataset construction and experimental process for developing of automated landmarking networks.

Figure 1 provides a visual representation of the entire workflow of this study, encompassing dataset creation and network utilization. The procedure of this research involved several key steps: preprocessing the image data; training and validating the CNN; and, finally, testing the CNN on a large-scale dataset.

### Dataset collection

This study used a dataset of 408 consecutive 3D facial images from adult patients, originally collected for assessing dentofacial deformities and planning orthognathic surgeries. These images were chosen irrespective of each patient’s sex, racial identity, type of malocclusion, or skeletal pattern but were based on specific research criteria: they had to be high-quality 3D facial images, without defects, of patients over 17 years old that had no significant facial hair. Patient consent was obtained regarding the use of these images for research purposes. This diverse dataset covers a wide range of facial characteristics relevant to orthognathic surgery, making it suitable for training and testing deep learning networks.

The images consisted of 68% preoperative scans, and the subjects had a mean age of 26.18 ± 8.6 years. Among the patients, 67% were female and 88% were of white ethnicity.

All 3D facial images were captured under a controlled and strict facial acquisition protocol, using passive stereo-photogrammetry of the Di3D imaging system (Dimensional Imaging, Hillington, Glasgow). The images were taken while the subjects had a neutral facial expression. The accuracy of this system has been evaluated previously, wherein an average system error of 0.21 mm was reported [14]. The captured facial images contained both texture and shape information and were saved in the ‘obj’ file format. All subjects’ identifying information was removed and an individual study code was assigned to each 3D facial image.

### Manual annotation of the anatomical landmarks

**Figure 2.**
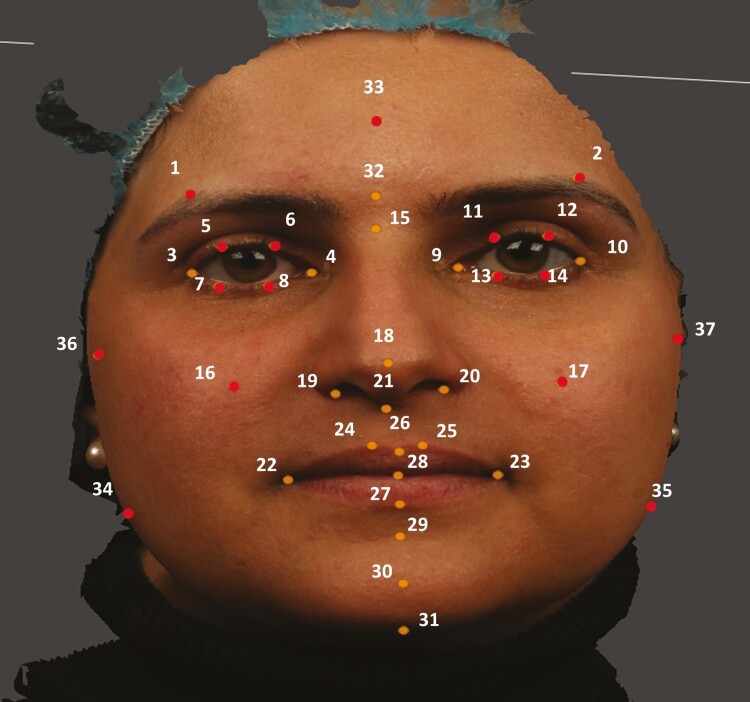
(A) Full set of landmarks indicators placed on 3D facial image by using Di3Dview software, (B) Zoom-in frontal view of 3D facial image. Orange dots represent primary landmarks. Red dots represent secondary landmarks.

Table 1 and Fig. 2 show the 37 landmarks that were manually digitized in this study [15–17]. A combination of both midline and peripheral points was included. The landmarks used in this study can be divided into two groups: primary and secondary landmarks. The primary landmarks are the key landmarks that are commonly used in clinical facial analysis studies and are more distinct, such as the corners of the lips and the tip of the nose. The secondary landmarks, on the other hand, are less distinct and are typically located between the primary landmarks: for example, on the cheeks. They play a more prominent role in increasing coverage and comprehensiveness in facial analyses. By using both primary and secondary landmarks, this study is able to present a more comprehensive collection of landmarks that accurately represent the facial structure and features of each patient.

**Table 1.** Names and definitions of landmarks used in this study.

Landmark number	Facial landmarks	Definition
**1,2**	**Superciliary point (right + left)**	The points located above most superior part of the eyebrows.
**3,10**	**Exocanthion**	Apex of the angle formed at the outer corner of the palpebral ﬁssure where the upper and lower eyelids meet.
	**(right + left)**	
**4,9**	**Endocanthion**	Apex of the angle formed at the inner corner of the palpebral ﬁssure where the upper and lower eyelids meet.
	**(right + left)**	
**5,6,11,12**	**Upper eyelid (right + left)**	Anchor points in the upper and lower eyelid.
**7,8,13,14**	**Lower eyelid (right + left)**	
**15**	**Nasion**	The midpoint on the soft tissue contour of the base of the nasal root where the frontal and nasal bones contact (nasofrontal suture).
**16,17**	**Cheek** ^*^	
	**(right + left)**	At the intersection between Camper’s plane and a line connecting the external eye canthus with the labial commissure. Camper’s plane is defined as passing through right and left tragus and subnasale landmarks.
**18**	**Pronasale (prn)**	Midline point marking the maximum protrusion of the nasal tip.
**19,20**	**Subalare (right + left)**	The point on the lower margin of the base of the nasal ala where the ala disappears into the upper lip skin.
**21**	**Subnasale**	Midpoint of the angle at the columella base where the lower border of the nasal septum and the surface of the upper lip meet (the apex of the nasolabial angle).
**22,23**	**Cheilion**	Point located at the corner of each labial commissure.
	**(right + left)**	
**24,25**	**Crista philtre**	The peak of Cupid’s bow.
	**(right + left)**	
**26**	**Labiale superius**	The midpoint of the vermilion line of the upper lip.
**27**	**Labiale inferius**	The midpoint on the vermilion line of the lower lip.
**28**	**Stomion**	Midpoint of the labial ﬁssure.
**29**	**Sublabiale**	Midpoint along the inferior margin of the cutaneous lower lip (labiomental sulcus).
**30**	**Pogonion**	The most anterior midpoint of the chin.
**31**	**Gnathion**	Midline point on the inferior border of the mandible.
**32**	**Glabella**	The most prominent midline point between the eyebrows, identical to bony glabella on the frontal bone.
**33**	**Metopion**	Most anterior (or most convex) midline point on the frontal bone. If the forehead region is relatively flat, place this landmark vertically at the midpoint between the superior facial border and glabella.
**34,35**	**Gonion (Right + Left)**	The most lateral point on the soft tissue contour of each mandibular angle located at the intersection of the tangent lines of the posterior border and the inferior border of the margin of the lower face.
**36,37**	**soft tissue zygion** ^**^	The soft tissue point located at each intersection of the lines orbitale—soft tissue porion and exocanthion—subaurale.

All landmark defined by (Farkas, 1994) except landmarks with * and **.

^*^Landmark defined by (Ferrario *et al*. 2003).

^**^Landmark defined by (Plooij *et al*. 2009).

The landmarks were identified on each 3D image using the Di3DView software. This software enabled us to simultaneously observe each 3D image from three different perspectives, allowing the image to be rotated and magnified. To assess the errors of the manual landmarking, 30 3D images were randomly selected and landmarked twice over a 2-week period by an experienced operator (an expert). Intra-operator error was evaluated using a paired Student’s *t*-test. A significance level of *P* < .0005 after Bonferroni correction was employed. Intra-class correlation coefficients were also calculated to determine the intra-operator reliability.

### The networks of the landmark detection framework

In this study, a patch-based CNN was employed for the purpose of 3D facial landmark detection. Instead of utilizing the entire facial image, patches surrounding individual landmarks within the 3D facial image were extracted and employed as inputs for the CNN. This approach involved the use of 2.5D patches, which encompass both texture and depth data from the local neighbourhood.

First, the 3D facial images were used to extract fixed-size square patches (40 mm × 40 mm) around the annotated landmarks, with each image having a dimension of 201 × 201 pixels. The landmark was centred within the extracted patch. Then, these patches were converted into a 2.5D representation, combining 2D texture and depth data for enhanced accuracy in facial landmark detection.

**Figure 3.**
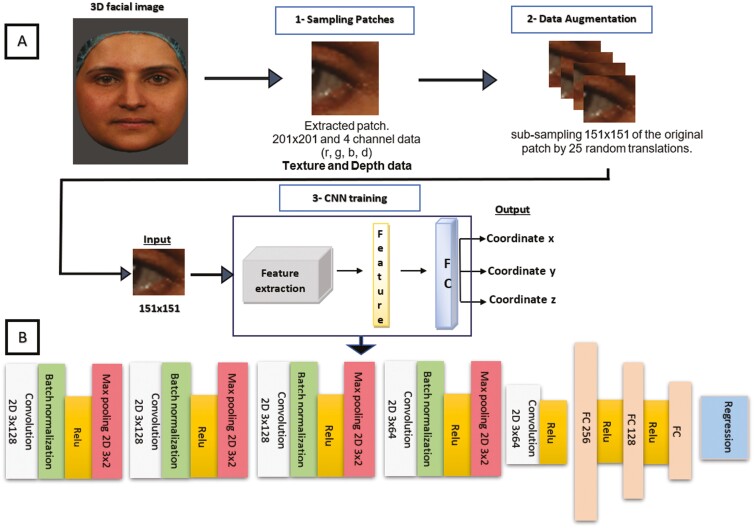
The framework of the Patch-based CNN for single landmark localization. (A) displaying the overall framework and (B) presenting the architecture of the landmark detection model.

An example of a 2.5D patch, centred at the nasal tip (prn), is illustrated in Fig. 3. This representation combines texture and depth information obtained by projecting the 3D surface onto a 2D plane, preserving both aspects. While computationally intensive, this process simplifies landmark detection for the network compared to handling a full 3D model, making it more efficient. By incorporating depth and 2D texture information (Red, Green, Blue), this 2.5D representation improves the landmark detection accuracy compared to traditional 2D methods and enhances the network’s understanding of facial 3D structures.

Data augmentation was carried out using translation cropping on 408 patches, resulting in a dataset of 10 200 PNG images (151 × 151 pixels) for each landmark. This expansion enabled us to capture image variations, reduced the dimensions of the data to improve the computational efficiency and helped the model recognize landmarks under diverse conditions, enhancing the CNN’s predictive accuracy for new data.

Paired with their corresponding landmark locations, these cropped sub-patches form a new dataset, which was used for training, validation, and testing. In total, 80% of the images were allocated for training (8160 images), and the remaining images were used for validation (10%, 1020 images) and testing (10%, 1020 images). The training and validation sets were employed to develop and refine the method, whereas the test sets were reserved for the final evaluation. It is worth mentioning that the test sets were not utilized during the training process.

**Figure 4.**
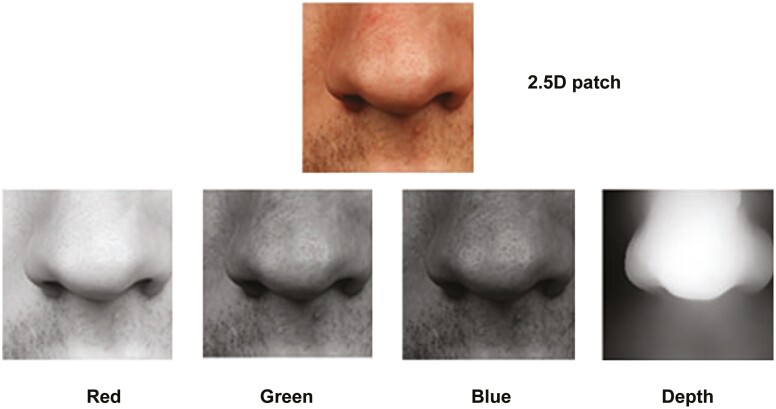
A 2.5D patch of the nasal tip.

Figure 4 summarizes the steps taken in this study for landmark detection with the patch-based CNN. For the deep learning of each landmark, we initially had 408 PNG images sized at 201 × 201, which were then augmented into 10 200 PNG images with a size of 151 × 151.

### System evaluation

The test dataset, comprising 1020 patches (10% of the main study dataset), was used to assess the performance of the developed networks. The accuracy of landmark localization was determined by comparing the automatically annotated landmarks with their manually annotated counterparts. This was carried out by directly comparing the coordinate values obtained from both methods.

The automated method was evaluated for each facial landmark (37 landmarks in total) by comparing the mean absolute distance in each of the three dimensions, i.e. by comparing the x-, y-, and z-axis coordinates between the manually digitized and automatically detected landmarks. In addition, the Euclidean distance was computed using the following formula:


$$\begin{array}{l} Distance=\sqrt{{\left(x1-x2\right)}^{2}+{\left(y1-y2\right)}^{2}+{\left(z1-z2\right)}^{2}} \end{array}$$

where x1, y1, and z1 are the coordinates for the manual detection, and x2, y2, and z2 are the coordinates for the automated detection. Descriptive statistics (mean error, standard deviation, and Euclidean distance) were compared between the manual and automated methods. A 95% conﬁdence interval was also estimated for the study outcomes.

The success detection rate (SDR, %) was calculated to measure the percentage of landmarks detected within a certain distance from their true positions. Predictions within 1mm of the manual identification result were considered clinically acceptable. The number of accurate identifications based on the SDR was divided into groups based on common ranges of ≤ 1.0 mm, 1.5 mm, 2.0 mm, and 3.0 mm.

## Results

### Manual landmark identification error

The overall mean of the intra-operator error, calculated across subjects, along all axes, and for all landmarks, was 0.56 ± 0.69 mm. These values ranged between 0.20 mm and 2.23 mm. Most of the landmark’s coordinates did not exhibit any statistically significant error based on the paired Student *t*-tests. The ICC was > 0.90, which indicates a high rate of reproducibility for intra-operator repetitive identiﬁcation.

### The accuracy of automated landmarking in comparison with the manually digitized ‘ground truth’

**Figure 5.**
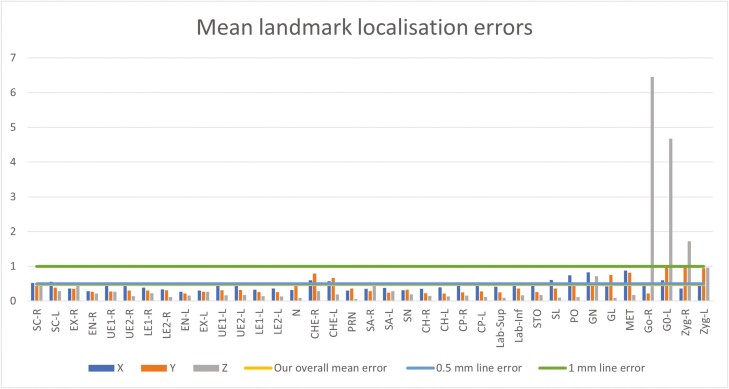
Landmark localization error of the CNN model for the 37 landmarks in each axis.

According to Fig. 5 and Supplementary Table 1, the overall mean of the accuracy along all axes for all landmarks was 0.47 ± 0.52 mm. The y-axis had the lowest mean error among all of the axes (0.41 ± 0.32 mm), while the x-axis had a higher mean error than the y-axis (0.45 ± 0.36 mm), and the z-axis had the highest mean error and standard deviation when compared to the other two axes (0.56 ± 0.89 mm).

The most accurately identified landmark was pronasale (z-axis), with a mean error of 0.06 mm. The identified landmark with the largest error was the right pogonion (z-axis), which had a mean error of 6.45 mm.

**Figure 6.**
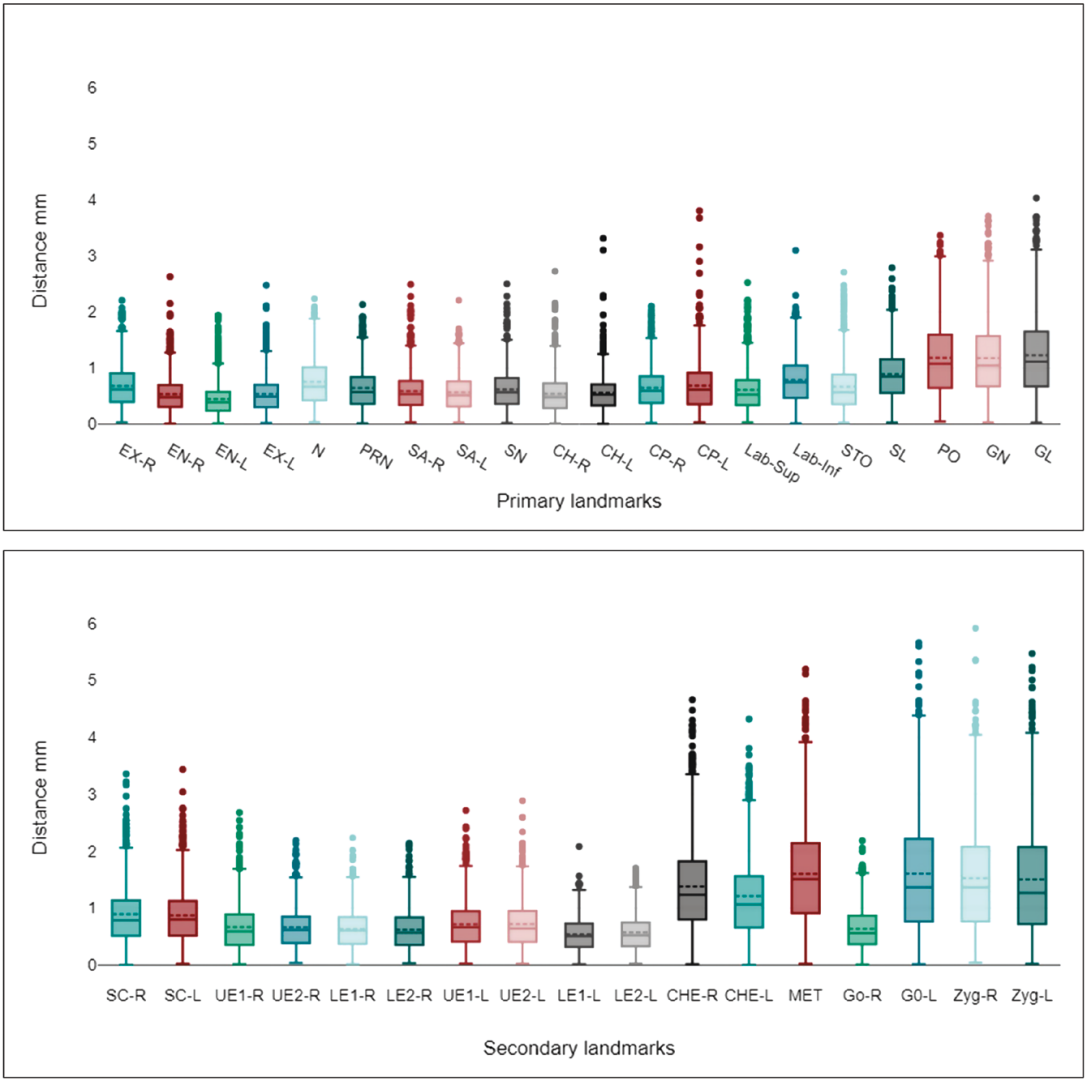
Box plot of localization errors for primary and secondary landmarks: assessing Euclidean distances between ground truth and automated CNN-based model estimations.

The landmark localization error was also calculated for each landmark by calculating the 3D Euclidean distance and the distribution of the error values for each landmark, as represented by the box plot in Fig. 6. The overall landmark localization error was 0.83 mm, with a standard deviation of 0.49 mm. The lowest localization error was noted at the corners of the eyes (endocanthion, exocanthion, R/L) and lips (cheilion, R/L). The results for the left gonion exhibited the largest discrepancy between the automated and manual landmarking, with a mean error of 1.61 mm and a standard deviation of 1.05 mm. Other landmarks that demonstrated errors of 1 mm or more included the right and left cheeks, the pogonion, the gnathion, the glabella, and the right and left zygion.

**Figure 7.**
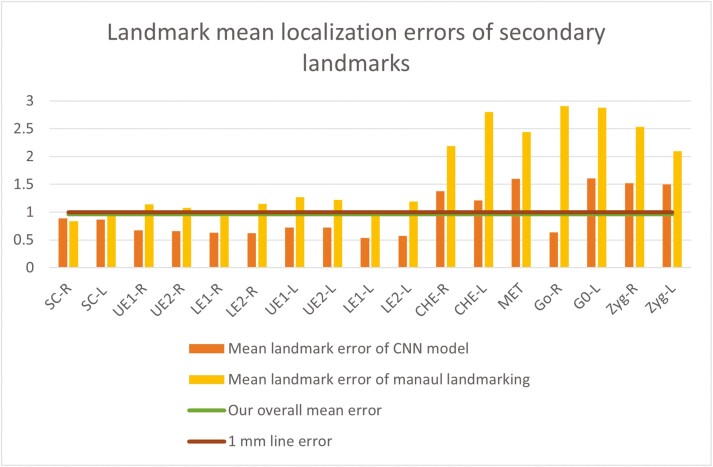
Comparison of mean localization errors between automated CNN model and manual landmarking method for primary (a) and secondary (b) landmarks.

It is evident from Fig. 7a that the primary landmarks generally exhibit low errors in automatic identification compared to the secondary landmarks. By comparing Fig. 6a and b, it can be observed that landmarks with higher errors in manual digitization also exhibit higher errors with the automated method.

As shown in Table 2, the right endocanthion achieved the highest SDR scores for each error range, with values of 94%, 99%, 100%, 100%, and 100%, respectively. The mean localization error for the right endocanthion was 0.54 ± 0.30 mm. On the other hand, the metopion had the lowest SDR scores for all error ranges, measuring 28%, 50%, 71%, 85%, and 93%, respectively. The mean localization error for the metopion was 1.6 ± 0.87 mm. Table 2 provides a comprehensive summary of the mean localization error values, the corresponding 95% confidence intervals, and the SDR values for each primary and secondary landmark obtained from the test data.

**Table 2.** The mean localization error and success detection rate (SDR) value of landmarks obtained from test data.

	Landmark	Success detection rate (SDR)%					Mean ± SD	95% Confidence interval of Mean
		1.0 mm	1.5 mm	2.0 mm	2.5 mm	3.0 mm		
**Primary landmarks**	EX-R	81	96	100	100	100	0.68 ± 0.39	0.65; 0.7
	EN-R	92	99	100	100	100	0.53 ± 0.31	0.51; 0.55
	**EN-L**	**94**	**99**	**100**	**100**	**100**	**0.45 ± 0.30**	**0.43; 0.46**
	EX-L	92	99	100	100	100	0.53 ± 0.32	0.51; 0.55
	N	75	92	99	100	100	0.75 ± 0.45	0.73; 0.78
	PRN	83	97	100	100	100	0.64 ± 0.37	0.62; 0.66
	SA-R	89	98	99	100	100	0.59 ± 0.34	0.56; 0.61
	SA-L	90	99	100	100	100	0.56 ± 0.32	0.55; 0.58
	SN	87	98	99	100	100	0.62 ± 0.32	0.59; 0.64
	CH-R	92	99	99	100	100	0.54 ± 0.34	0.52; 0.56
	CH-L	93	99	99	100	100	0.55 ± 0.32	0.53; 0.57
	CP-R	85	98	100	100	100	0.64 ± 0.36	0.62; 0.66
	CP-L	80	95	99	100	100	0.68 ± 0.44	0.66; 0.71
	Lab-Sup	86	97	99	100	100	0.61 ± 0.38	0.58; 0.63
	Lab-Inf	72	95	99	100	100	0.79 ± 0.42	0.76; 0.81
	STO	81	95	98	100	100	0.66 ± 0.43	0.64; 0.69
	SL	63	92	98	100	100	0.89 ± 0.44	0.86; 0.91
	PO	46	71	87	95	99	1.18 ± 0.68	1.14; 1.22
	GN	47	72	87	96	98	1.17 ± 0.69	1.13; 1.21
	GL	44	69	83	94	98	1.22 ± 0.73	1.18; 1.27
Overall primary landmarks		79	93	97	99	100	0.72 ± 0.42	
**Secondary landmark**	SC-R	68	87	95	99	100	0.89 ± 0.54	0.86; 0.92
	SC-L	66	90	97	99	100	0.87 ± 0.48	0.84; 0.9
	UE1-R	81	96	99	100	100	0.67 ± 0.41	0.64; 0.7
	UE2-R	83	98	100	100	100	0.66 ± 0.36	0.64; 0.69
	LE1-R	87	99	100	100	100	0.63 ± 0.34	0.61; 0.65
	LE2-R	87	99	99	100	100	0.62 ± 0.35	0.6; 0.64
	UE1-L	78	96	99	100	100	0.72 ± 0.4	0.69; 0.74
	UE2-L	78	94	99	100	100	0.72 ± 0.42	0.69; 0.75
	LE1-L	93	100	100	100	100	0.54 ± 0.29	0.52; 0.56
	LE2-L	88	99	100	100	100	0.57 ± 0.33	0.55; 0.59
	CHE-R	37	62	81	92	96	1.38 ± 0.79	1.33; 1.43
	CHE-L	46	72	84	92	98	1.21 ± 0.74	1.16; 1.25
	MET	28	50	71	85	93	1.60 ± 0.87	1.54; 1.65
	Go-R	84	98	100	100	100	0.64 ± 0.37	0.61; 0.66
	G0-L	35	55	69	81	89	1.61 ± 1.05	1.54; 1.67
	Zyg-R	36	55	73	83	91	1.52 ± 0.96	1.46; 1.58
	Zyg-L	37	58	73	84	91	1.5 ± 0.99	1.44; 1.56
Overall secondary landmarks		65	82	90	94	97	0.96 ± 0.57	
All landmarks		72	88	94	97	99	0.83 ± 0.49	

Primary landmarks: EX-R: Exocanthion (right), EN-R: Endocanthion (right), EN-L: Endocanthion (left), EX-L: Exocanthion (left), N: Nasion, PRN: Pronasale, SA-R: Subalare (right), SA-L: Subalare (left), SN: Subnasale, CH-R: Cheilion (right), CH-L: Cheilion (left), CP-R: Crista philtre (right), CP-L: Crista philtre (left), Lab-Sup: Labiale superius, Lab-Inf: Labiale inferius, STO: Stomion, SL: Sublabiale, PO: Pogonion, GN: Gnathion, GL: Glabella.

Secondary landmarks: SC-R: Superciliary point (right), SC-L: Superciliary point (left), UE1-R: Upper eyelid (right), UE2-R: Upper eyelid (right), LE1-R: Lower eyelid (right), LE2-R: Lower eyelid (right), UE1-L: Upper eyelid (left), UE2-L: Upper eyelid (left), LE1-L: Lower eyelid (left), LE2-L: Lower eyelid (left), CHE-R: Cheek (right), CHE-L: Cheek (left), MET: Metopion, Go-R: Gonion (Right), Go-L: Gonion (Left), Zyg-R: soft tissue zygion (Right), Zyg-L: soft tissue zygion (Left).

SD: standard deviation, CI: confidence interval. Landmarks with red highlight indicates a mean error > 1 mm. Bold indicates lowest localization error.

Table 3 displays the results reported in the literature, revealing a noteworthy difference between the results reported in prior studies and those of the current study.

**Table 3.** Comparison of our proposed model with previously published automated landmarking model in clinical and biological literature.

Method	No. LM	Overall mean error (mm)	Accuracy rate: % of correctly detected landmarks based on MAD within defined thresholds			
			1.0 mm	1.5 mm	2.0 mm	3.0 mm
(Guo *et al.* 2013) [18]	17	1.39 ± 0.97	12%	82%	88%	100%
(Liang *et al*. 2013) [19]	20	2.64	10%	15%	40%	65%
(Sukno *et al*. 2014) [20]	14	2.3	0%	33%	71%	79%
(De Jong *et al*. 2018) [21]	21	1.7 ± 0.4	0%	0%	33%	71%
(Vezzetti *et al.* 2018) [22]	13	4.73	0%	0%	0%	8%
(Abu *et al*. 2019) [23]	10	2.23	40%	60%	60%	60%
(Wang *et al*. 2019) [24]	23	2.23	0%	0%	13%	35%
(Bannister *et al*. 2020) [25]	12	2.5 ± 2.0	0%	0%	8%	83%
(Baksi *et al*. 2021) [26]	21	3.2 ± 1.64	0%	0%	24%	38%
(Zhang *et al.* 2023) [27]	32	2.62 ± 2.39	0%	0%	25%	53%
**Proposed method**	**37**	**0.83 ± 0.49**	**76%**	**95%**	**100%**	**100%**

LM, landmark; MAD, mean absolute difference; No, number.

## Discussion

The results of this study show that the proposed CNN-based approach surpassed other existing automatic models in detecting 3D landmarks on human faces in a clinical setting. This was achieved by including a large sample size, which led to a greater number of detected landmarks and significantly reduced the localization error. In a very recent study conducted by [27], 32 landmarks were automatically annotated. However, their reported mean error distance for all 32 landmarks was 2.62 mm (SD, 2.39 mm).

In this study, low localization errors were achieved through a comprehensive CNN-training approach that combined texture and depth information. This proposed approach allowed the model to leverage both texture-based and shape-based analyses, resulting in improved accuracy. In addition, standardized high-quality images from a clinical database minimized the variability and confounding factors, further enhancing the model’s accuracy. Ensuring consistent acquisition conditions, lighting, facial expressions, and backgrounds across the images can effectively reduce the variability and confounding factors that might affect the performance of the automated landmarking model [28].

This study revealed that automated identification of midline landmarks was more accurate than that of lateral (peripheral) landmarks. This finding aligns with previous studies on both automated and manual landmark identification. Peripheral landmarks are usually located in areas that lack distinct anatomical features, which poses a challenge for automated algorithms to accurately detect landmarks in those regions. Similarly, a study by Torres *et al.* [29] found a limitation of their developed automated model when detecting landmarks located at non-featured and ﬂat regions.

In contrast to the findings of [25] and [26], who reported poor performance in automated landmarking at the corners of the eyes, this study demonstrated a significant improvement in the accuracy of these specific landmarks as well as the cheilion landmarks (R/L) using the automated CNN-based landmarking approach. This improvement is attributed to the reliable ground-truth data used and the unique features of these landmarks. A CNN model can automatically extract significant features without any human supervision, enhancing precision and enabling automation [30]. Our method effectively exploits these features, resulting in higher accuracy, particularly for primary landmarks (Figs 5 and 6).

The quality of the annotated ground-truth data significantly impacts a CNN model’s performance [31]. Inconsistencies in annotations within the training set present a challenge for CNN models to effectively learn and generalize new data, leading to errors in landmark detection. In this study, the relationship between the accuracy of automated CNN models in detecting specific landmarks and the accuracy and consistency of the manual landmarking method (intra-operator error) was examined. It was found that landmarks identified with higher errors in the manual landmarking approach also exhibited higher errors when the automated landmarking method was used (Fig. 6). This was particularly evident in the zygion landmarks, which showed the highest error in the z-axis direction. This can be attributed to the fact that the zygion occupies a flat area with ill-defined features, as well as inconsistencies in annotations within the training set, which lead to errors in landmark detection. Improvements are necessary to address the limited capability of patch-based CNNs in localizing landmarks with ill-defined features. One potential solution for this could involve incorporating a hybrid approach that combines a patch-based CNN with other techniques or models.

The presence of image artefacts caused by hair and the presence of reflective objects can reduce the quality of the peripheral areas of facial 3D images [32, 18] identified the pogonion and earlobes as particularly challenging landmarks to automatically locate, which can be attributed to the strong influence of facial or head hair, potentially leading to larger mean errors and standard deviations when using an automated detection method. In this study, the gonion was landmarked with low precision, and this might be due to the quality of the 3D images in this area. Consequently, localizing landmarks in peripheral regions is considerably more challenging and contributes to the observed low precision and consistency in identifying certain landmarks.

It was observed that there was a difference in the automated accuracy patterns between bilaterally positioned landmarks, specifically the right and left gonion. This could be attributed to shadowing or variations in lighting conditions between the two sides, which may affect the precision of the landmark identification.

To ensure accurate automatic landmarking, it is vital to assess the reproducibility of manual landmarking. Many studies on landmark detection for 3D facial images lack this crucial information [11]. To enhance people’s confidence in automatic landmarking algorithms, researchers should follow recognized reporting standards, document the landmark annotation process, and implement quality control measures [33, 34]. In this study, the intra-operator reproducibility for each landmark was assessed along three axes. These results validate the examination techniques used and ensure the validity of our study.

This study presents advancements in automatic landmark detection for 3D facial images within clinical contexts, offering potential applications in orthodontics, orthognathic surgery, and craniofacial research. The accurate placement of anatomical landmarks is critical for meeting clinical standards and enabling the precise identification of facial measurements. Moreover, the utilization of dense correspondence analysis (comprehensive surface analysis) techniques facilitates comparisons between patients’ facial features and those of unaffected individuals, thereby enhancing clinical assessments and providing valuable insights into treatment efficacy and patient outcomes. Furthermore, the implementation of accurate landmarking streamlines the workflow of 3D facial image-processing pipelines, particularly benefitting genetic and developmental studies where detailed phenotyping is essential for investigating genetic influences on facial morphology.

While this study represents progress in automatic landmark detection for 3D facial images in clinical settings, it is crucial to acknowledge its several limitations. These include its reliance on data from a single centre, the lack of external validation, and the use of a single annotator. Future research should incorporate diverse datasets from multiple centres, patients of various ethnicities, and multiple annotators to validate the model’s effectiveness across different populations. External validation using diverse image captures, imaging protocols, and equipment is also essential. In addition, the patch-based CNN approach has certain drawbacks, such as its time consumption and the absence of global context information. A potential solution for these issues could be found with a hybrid approach that combines a patch-based CNN with other techniques. Future studies should aim to overcome these limitations.

## Conclusion

The automated landmarking method utilized in this study demonstrated accurate landmark detections comparable to those obtained manually by an observer (the ground truth).

This study makes a significant contribution to the field of detecting landmarks within 3D facial images by demonstrating the effectiveness of using CNNs in clinical settings. Our approach, a patch-based method, involves training a CNN model using augmented patches based on expert-established ground-truth data. Ultimately, 37 soft-tissue facial landmarks were localized, with an overall mean error of 0.83 ± 0.49 mm; these findings strongly support this method’s effectiveness in landmark detection. This could aid in diagnosing dentofacial deformities, as well as in genetic and developmental studies that rely on large datasets. Future research should focus on enhancing the model’s robustness and broadening this study’s scope to include other population groups.

## Supplementary Material

cjae056_suppl_Supplementary_Tables_1

## Data Availability

The data underlying this article are available in the article and in its online supplementary material.
