# Supplementary material for: Patch-based convolutional neural networks for automatic landmark detection of 3D facial images in clinical settings
Source: Eur J Orthod. 2024 Nov 28;46(6):cjae056. doi: 10.1093/ejo/cjae056 (PMC11602742; doi:10.1093/ejo/cjae056)
Supplement: cjae056_suppl_Supplementary_Tables_1 [file cjae056_suppl_supplementary_tables_1.docx]

| **Landmarks**  Supplementary Table 1: The mean error of the proposed CNN landmark model both in terms of each axis and as the Euclidean distance. | | | **Mean Absolute differences** | | | | | | **Euclidean distances Mean** | **SD** |
| --- | --- | --- | --- | --- | --- | --- | --- | --- | --- | --- |
|  |  |  | **X** | | **Y** | | **Z** | |  |  |
|  |  |  | **m** | **SD** | **m** | **SD** | **m** | **SD** |  |  |
| 1 | **Superciliary point (right)** | **SC-R** | 0.52 | 0.46 | 0.44 | 0.33 | 0.44 | 0.38 | 0.89 | 0.54 |
| 2 | **Superciliary point (left)** | **SC-L** | 0.56 | 0.43 | 0.39 | 0.32 | 0.29 | 0.24 | 0.87 | 0.48 |
| 3 | **Exocanthion (right)** | **EX-R** | 0.36 | 0.29 | 0.35 | 0.27 | 0.51 | 0.62 | 0.68 | 0.39 |
| 4 | **Endocanthion (right)** | **EN-R** | 0.29 | 0.24 | 0.27 | 0.21 | 0.22 | 0.19 | 0.53 | 0.31 |
| 5 | **Upper eyelid (right)** | **UE1-R** | 0.45 | 0.37 | 0.28 | 0.24 | 0.27 | 0.26 | 0.67 | 0.41 |
| 6 | **Upper eyelid (right)** | **UE2-R** | 0.43 | 0.33 | 0.30 | 0.23 | 0.14 | 0.16 | 0.66 | 0.36 |
| 7 | **Lower eyelid (right)** | **LE1-R** | 0.39 | 0.30 | 0.30 | 0.22 | 0.23 | 0.20 | 0.63 | 0.34 |
| 8 | **Lower eyelid (right)** | **LE2-R** | 0.34 | 0.26 | 0.31 | 0.25 | 0.12 | 0.12 | 0.62 | 0.35 |
| 9 | **Endocanthion (left)** | **EN-L** | 0.27 | 0.22 | 0.22 | 0.20 | 0.16 | 0.16 | 0.45 | 0.30 |
| 10 | **Exocanthion (left)** | **EX-L** | 0.30 | 0.26 | 0.27 | 0.21 | 0.27 | 0.27 | 0.53 | 0.32 |
| 11 | **Upper eyelid (left)** | **UE1-L** | 0.47 | 0.36 | 0.31 | 0.25 | 0.17 | 0.17 | 0.72 | 0.40 |
| 12 | **Upper eyelid (left)** | **UE2-L** | 0.47 | 0.35 | 0.32 | 0.27 | 0.18 | 0.17 | 0.72 | 0.42 |
| 13 | **Lower eyelid (left)** | **LE1-L** | 0.33 | 0.24 | 0.26 | 0.2 | 0.14 | 0.12 | 0.54 | 0.29 |
| 14 | **Lower eyelid (left)** | **LE2-L** | 0.36 | 0.28 | 0.26 | 0.22 | 0.13 | 0.13 | 0.57 | 0.33 |
| 15 | **Nasion** | **N** | 0.32 | 0.26 | 0.43 | 0.33 | 0.09 | 0.13 | 0.75 | 0.45 |
| 16 | **Cheek (right)** | **CHE-R** | 0.60 | 0.48 | 0.79 | 0.57 | 0.29 | 0.28 | 1.38 | 0.79 |
| 17 | **Cheek (left)** | **CHE-L** | 0.57 | 0.51 | 0.67 | 0.52 | 0.19 | 0.17 | 1.21 | 0.74 |
| 18 | **Pronasale** | **PRN** | 0.30 | 0.24 | 0.36 | 0.27 | 0.06 | 0.10 | 0.64 | 0.37 |
| 19 | **Subalare (right)** | **SA-R** | 0.35 | 0.26 | 0.29 | 0.23 | 0.47 | 0.47 | 0.59 | 0.34 |
| 20 | **Subalare (left)** | **SA-L** | 0.38 | 0.31 | 0.24 | 0.19 | 0.29 | 0.29 | 0.56 | 0.32 |
| 21 | **Subnasale** | **SN** | 0.31 | 0.24 | 0.33 | 0.26 | 0.19 | 0.19 | 0.62 | 0.35 |
| 22 | **Cheilion (right)** | **CH-R** | 0.35 | 0.29 | 0.23 | 0.21 | 0.15 | 0.19 | 0.54 | 0.34 |
| 23 | **Cheilion (left)** | **CH-L** | 0.4 | 0.3 | 0.22 | 0.18 | 0.13 | 0.13 | 0.55 | 0.32 |
| 24 | **Crista philtre (right)** | **CP-R** | 0.46 | 0.36 | 0.25 | 0.2 | 0.16 | 0.19 | 0.64 | 0.36 |
| 25 | **Crista philtre (left)** | **CP-L** | 0.47 | 0.39 | 0.28 | 0.26 | 0.12 | 0.13 | 0.68 | 0.44 |
| 26 | **Labiale superius** | **Lab-Sup** | 0.41 | 0.35 | 0.25 | 0.22 | 0.09 | 0.13 | 0.61 | 0.38 |
| 27 | **Labiale inferius** | **Lab-Inf** | 0.5 | 0.38 | 0.36 | 0.27 | 0.17 | 0.17 | 0.79 | 0.42 |
| 28 | **Stomion** | **STO** | 0.47 | 0.41 | 0.26 | 0.22 | 0.18 | 0.19 | 0.66 | 0.43 |
| 29 | **Sublabiale** | **SL** | 0.61 | 0.43 | 0.36 | 0.28 | 0.10 | 0.12 | 0.89 | 0.44 |
| 30 | **Pogonion** | **PO** | 0.74 | 0.56 | 0.53 | 0.46 | 0.12 | 0.18 | 1.18 | 0.68 |
| 31 | **Gnathion** | **GN** | 0.83 | 0.67 | 0.45 | 0.39 | 0.72 | 0.77 | 1.17 | 0.69 |
| 32 | **Glabella** | **GL** | 0.42 | 0.36 | 0.75 | 0.55 | 0.09 | 0.12 | 1.22 | 0.73 |
| 33 | **Metopion** | **MET** | 0.88 | 0.63 | 0.82 | 0.63 | 0.18 | 0.20 | 1.60 | 0.87 |
| 34 | **Gonion (Right)** | **Go-R** | 0.48 | 0.39 | 0.22 | 0.17 | 6.45 | 12.33 | 0.64 | 0.37 |
| 35 | **Gonion (Left)** | **Go-L** | 0.60 | 0.45 | 1.01 | 0.74 | 4.68 | 7.13 | 1.61 | 1.05 |
| 36 | **soft tissue zygion (Right)** | **Zyg-R** | 0.36 | 0.29 | 1.00 | 0.72 | 1.72 | 5.23 | 1.52 | 0.96 |
| 37 | **soft tissue zygion (Left)** | **Zyg-L** | 0.45 | 0.41 | 0.95 | 0.73 | 0.96 | 0.88 | 1.50 | 0.99 |
| **Average** | | | **0.45** | **0.36** | **0.41** | **0.32** | **0.56** | **0.89** | **0.83** | **0.49** |
| **Max** | | | **0.88** | **0.67** | **1.01** | **0.74** | **6.45** | **12.33** | **1.61** | **1.05** |
| **Min** | | | **0.27** | **0.22** | **0.22** | **0.17** | **0.06** | **0.10** | **0.45** | **0.29** |
